# Amantadine in Treatment of Dysthymia—The Pilot Case Series Study

**DOI:** 10.3390/ph16060897

**Published:** 2023-06-19

**Authors:** Marek Krzystanek, Ewa Martyniak, Artur Pałasz, Katarzyna Skałacka, Artur Chwalba, Piotr Wierzbiński

**Affiliations:** 1 Department and Clinic of Psychiatric Rehabilitation, Faculty of Medical Sciences, Medical University of Silesia in Katowice, 40-635 Katowice, Poland; evamartyniak@gmail.com; 2 Department of Histology, Faculty of Medical Sciences, Medical University of Silesia in Katowice, 40-752 Katowice, Poland; artiassone@gmail.com; 3 Institute of Psychology, University of Opole, 45-040 Opole, Poland; katarzyna.skalacka@uni.opole.pl; 4 Pharmacology Department, Faculty of Medical Sciences in Zabrze, Medical University of Silesia in Katowice, 41-808 Zabrze Rokitnica, Poland; artur.adam.chwalba@gmail.com; 5 Psychiatry Outpatient Clinic, 91-498 Lodz, Poland; piotr1wierzbinski@gmail.com

**Keywords:** dysthymia, persistent depressive disorder, amantadine, treatment failure, off-label treatment

## Abstract

Dysthymia is a common chronic mood disorder in which isolated symptoms of depression persist for at least 2 years. Despite the many medications recommended for the treatment of dysthymia, no recommendations have yet been made for the treatment of patients who fail to achieve clinical improvement. This justifies attempts to identify second-line drugs for the treatment of dysthymia. In an open and naturalistic case study, five patients diagnosed with dysthymia in whom at least one antidepressant treatment was ineffective were treated with amantadine. In the age- and gender-matched external control group, patients were treated with sertraline at 100 mg/day. Depressive symptoms were assessed using HDRS-17. Two men and three women were treated with 100 mg amantadine for 3 months with 3–5 months follow-up. After 1 month of treatment with amantadine, a significant reduction in the intensity of depressive symptoms was achieved in all patients, and the clinical improvement increased over the next 2 months of treatment. No deterioration in well-being was observed in any patient after discontinuation of amantadine. The effect of amantadine treatment was comparable to that of sertraline treatment in patients with dysthymia who improved with this drug. The present study indicates that amantadine is an effective and well-tolerated drug in the treatment of dysthymia. Amantadine may be associated with a quick improvement in symptoms in the treatment of dysthymia. Treatment with this drug seems to be associated with good tolerability and persistency of the therapeutic effect after the discontinuation of the treatment.

## 1. Introduction

Dysthymia is a chronic mood disorder where single depressive symptoms persist for at least two years. Lifetime prevalence of dysthymia ranges from 2.1 to 6.4% [1]. Even though the symptoms do not meet the criteria of major depressive episode, their persistence for many years causes a deterioration of the patients’ well-being and significantly affects their quality of life. The clinical construct of dysthymia is often placed on the spectrum between personality style and persistent depressive disorder; however, clinical studies indicate biological rather than psychological causes [2].

Dysthymia combines high chronicity of chronic major depression or double depression with low severity of episodic major depression [2]. Dysthymia often coexists with major depression, which is referred to as double depression [3]. A new clinical construct was introduced in the DSM-5 classification—persistent depressive disorder, covering dysthymia, double depression, and chronic major depressive episodes. The inclusion of dysthymia in a group with persistent depressive disorder, however, may blur the diagnostic boundaries of dysthymia itself and lead to the loss of its autonomic clinical picture. Daily practice shows that episodes of dysthymia occur independently in patients and that their treatment can be a difficult clinical challenge.

Both pharmacological and psychotherapeutic treatments are effective in treating dysthymia, with a synergistic effect when both forms of treatment are combined [4]. Overall, the pharmacological treatment of dysthymia is efficacious and well tolerated. Among many clinically tested antidepressants, many agents have demonstrated higher efficacy compared with the placebo. These include paroxetine, sertraline, fluoxetine, moclobemide, and imipramine, with a lack of convincing evidence in the cases of duloxetine and desvenlafaxine [4]. Of all of the antipsychotic drugs for the treatment of dysthymia, the efficacy of small doses of amisulpride [5] has so far been confirmed.

Therapeutic recommendations for the pharmacological treatment of dysthymia do not provide an appropriate path to follow in the event of treatment failure [4]. This article collects five cases of treatment of dysthymia in which at least one previous treatment was ineffective. Moreover, the presented cases of treatment failure dysthymia were treated with amantadine, which may have an antidepressant effect. The antidepressant effect of amantadine has been known since 1971 and was subsequently confirmed in the following years [6,7,8,9,10,11,12]. According to the authors’ knowledge, this is the first report on the effectiveness of amantadine in the treatment of sub-depressive mood disorder, also known as dysthymia.

## 2. Results

In the study group, adding amantadine to treatment significantly reduced the dysthymia symptoms reported in the HDRS-17 scale in all patients [F(3, 12) = 75.19; *p* < 0.001; eta2 = 0.95]. The additional analysis excluded gender differences between patients [F(1, 3) = 0.01; *p* = 0.913; eta2 = 0.01]. The improvement in all patients (regardless of their gender) between the first and the last visit was also significant [ΔM = 6.0; SD = 1.0; 95%CI(4.76–7.24); t(4) = 13.42; *p* < 0.001; d = 1.0]. Table 1 presents the results in individual score of the patients’ HDRS-17 scale in the study group at subsequent visits.

The tests of between-subjects effects has shown that control group is statistically different from the study group [F(1, 8) = 5.90; *p* = 0.041; eta2 = 0.43], but interaction effect of group and subsequent visit is statistically insignificant [F(3, 8) = 1.39; *p* = 0.27; eta2 = 0.15]. The tests of within-subject’s contrasts are also statistically insignificant. This result suggests that the effects of amantadine use are no different from standard dysthymia treatment. Individual scores of the patients’ HDRS-17 scale at subsequent visits in the amantadine-treated study group and the sex- and age-matched sertraline-treated control group are presented in Table 2.

Table 3 shows changes in symptoms in individual HDRS-17 items. In each case, there is a clear decrease in the intensity and gradual disappearance of individual depressive symptoms in patients after the initiation of amantadine treatment. After an average of 3 months of treatment (V4), no previously persistent symptoms were found in any of the patients.

### Individual Case Reports Are Summarized Below

Case 1. A 34-year-old man. In 2016, he had one reactive depressive episode related to financial problems and excessive workload. It was then overlapped by the distress of the patient’s mother’s illness, who died two years later. During that depressive episode, the main symptoms were anhedonia, apathy, lack of energy, and tearfulness. He then periodically took opipramol up to 150 mg/d due to anxiety and sleep problems. After his mother died, he did not fully recover, and he did not feel well after. In 2020, he came to a mental health clinic for chronic tearfulness, loss of interest, anhedonia, and decreased sexual pleasure as well as decreased libido. The patient then received escitalopram at a dose of 10 mg/day. After one month, the dose was increased to 15 mg due to the lack of improvement. As no improvement was still seen in the following 4 weeks, the drug was switched to sertraline, first at 50 mg/day for 6 days, and then 100 mg/day. The predominant symptom of the patient was still a lack of pleasure and apathy. Due to the lack of improvement, after 6 weeks of treatment he was offered amantadine at a dose of 100 mg/d, to which he agreed. The HDRS-17 was performed. After a month, at the next outpatient visit, the patient reported a reduction in anhedonia and ceased to be tearful. The patient said: “I stopped crying”. No depressive symptoms also were observed in the patient in the next two visits. The patient took amantadine for 6 months, then discontinued it. Recurrence of dysthymia symptoms was not observed. During the entire period of amantadine treatment, the patient did not report any side effects.

Case 2. A 38-year-old woman was not previously treated psychiatrically. She reported to an outpatient mental health clinic in 2019 due to chronic apathy and the impression that she did not feel emotions and that she was indifferent to everyday matters, and non-responsive to her husband and even children. She said that actually “I feel like I’m dead”, and “I’m faking emotional reactions, but I don’t feel anything”. She said that she saw no point in this life continuing and she dreamed of a natural catastrophe that would end her life. She had no suicidal thoughts or tendencies. In addition to anhedonia and thoughts of resignation, she complained of a slowdown in activity since she did not want anything, and a weakened libido. She said that she had sex with her husband only for his sake, and she herself did not feel any sexual desire. She received sertraline at the target dose of 100 mg/day. After one month, the dose was increased to 150 mg/day due to no improvement. As the patient still did not feel any change in her well-being after the next 6 weeks, she was offered to take amantadine 100 mg in the morning. After a month, the patient reported better motivation to act and improved libido. She said she was starting to feel feelings. At the third visit, the severity of anhedonia decreased, and after 3 months the patient did not report any decrease in pleasure. After 6 months, she discontinued the drug, and no recurrence of the dysthymia symptoms was observed. During the treatment with amantadine, the patient did not report any side effects of the drug.

Case 3. A woman at the age of 42. At 22, she had an episode of postpartum depression, when she took fluoxetine, after which she returned to normal functioning. Recently, she visited an outpatient mental health clinic because of a negative change in her functioning that lasted for 4–5 years. She complained of tearfulness and cried for no reason, with attempts to hide it. In her subjective report anhedonia was the most severe symptom, and she also complained of a lack of desire and loss of orgasm. The patient received fluoxetine 20 mg in the morning. As there was no improvement, the dose was increased to 30 mg after one month, and to 40 mg daily after another month. Still there was no improvement. Then, the fluoxetine was gradually withdrawn, and the patient received tianeptine at a dose of 37.5 mg/day. Since there was still no improvement in her well-being, after 6 weeks the patient was offered to take amantadine 100 mg in the morning. The patient gave her consent. After a month, she reported a significant improvement in the feeling of pleasure and an improvement in libido. The tearfulness was gone, too. At subsequent visits, the patient had no depressive symptoms anymore. Because after discontinuing amantadine after 6 months, she had a feeling of worsening mood, she took it for another 2 months and stopped the drug. The patient admitted that “I think I got scared of drug discontinuation” and there was no return of depressive symptoms then. The patient said: “I live normally again; I did not think it was so pleasant”. There were no side effects of amantadine throughout the whole treatment period.

Case 4. A woman aged 37. She visited an outpatient mental health clinic in 2019 after several previous treatment attempts. She had previously taken venlafaxine at a dose of 225 mg/day, then bupropion 150 mg/day, then citalopram 40 mg/day, and recently moclobemide 450 mg/day. No improvement was observed to any of those drugs. She had stopped taking moclobemide six months earlier after 4 months of treatment and continued to feel unwell. The symptoms were dominated by anhedonia, tearfulness, and a lack of will to live (without suicidal thoughts or tendencies). She complained of a weakened sexual desire and a feeling of general breakdown and weakness. She was offered to take amantadine at a dose of 100 mg/day. At the next visit, on her subjective report all depressive symptoms disappeared. She said: “It’s like a miracle, I haven’t felt so well for a long time”. However, the assessment with the HDRS-17 revealed still a mild severity of anhedonia. The patient reported that the improvement occurred within the first week of treatment. No depressive symptoms were observed at subsequent visits. The patient discontinued the treatment after 6 months, and no deterioration in well-being was observed after discontinuation of amantadine. The treatment with amantadine did not cause any side effects in the patient.

Case 5. A 44-year-old man. In 2021, he visited a psychiatrist for the first time in his life. The examination revealed single depressive symptoms: anhedonia, decreased interest, and decreased libido. The patient said that these symptoms had lasted 5–7 years before, but within the last year he stopped feeling any pleasure and he lost interest in anything. He said he was playing a normal person: “everything looks fine to my family, but it isn’t”. His day-to-day functioning was undisturbed, and his quality of functioning was much below his expectations. He was proposed to be treated with sertraline, but he refused to take any SSRI due to his libido problems. He agreed to start taking moclobemide at the target dose of 450 mg/d. After 2 months, he felt some improvement in mood, but assessed it only by 20–30%. He was offered treatment with amantadine at a dose of 100 mg/day. When he came back for a visit 5 weeks later, he said there was at least a 50% improvement in his pleasure experience. After another month, he did not report any depressive symptoms, but on the next visit he again complained of a reduction in his sexual desire, as confirmed by the patient’s wife who accompanied him. Due to complaints of decreased libido, amantadine was administered for 8 months. After that period the patient admitted, however, that he did not feel sexual desire for his wife but was having a love affair and that he did not complain of sexual dysfunction in the relationship with his lover. No side effects occurred during treatment with amantadine, and no deterioration in well-being was observed after its discontinuation.

## 3. Discussion

The analysis of clinical trials, meta-analyzes and clinical recommendations regarding the treatment of dysthymia shows many therapeutic options in the treatment of this chronic mood disorder [13]. However, no recommendations were identified specifically for individuals with dysthymia or subthreshold or persistent minor depression who had failed previous treatment [13]. This indicates the need to look for drugs that may be drug of choice in the case of failed or inadequate response in the treatment of dysthymia. Our case series study may indicate that amantadine is a candidate for such a drug that is effective in dysthymic patients for whom previous treatment has failed.

First-line medications for dysthymia also may have side effects [4] and a switch to another medication is required. If it is a drug from the group of selective serotonin reuptake inhibitors, the change to another drug from this group must consider wash-out, which is to prevent undesirable serotonin symptoms. In such a situation, amantadine, as a drug with a different mechanism of action, may be an alternative to SSRIs. It is worth adding that in our study, amantadine was effective in low doses and the treatment tolerance was very good. After introducing amantadine into the treatment, no side effects were observed in any of the studied patients.

**Figure 1:**
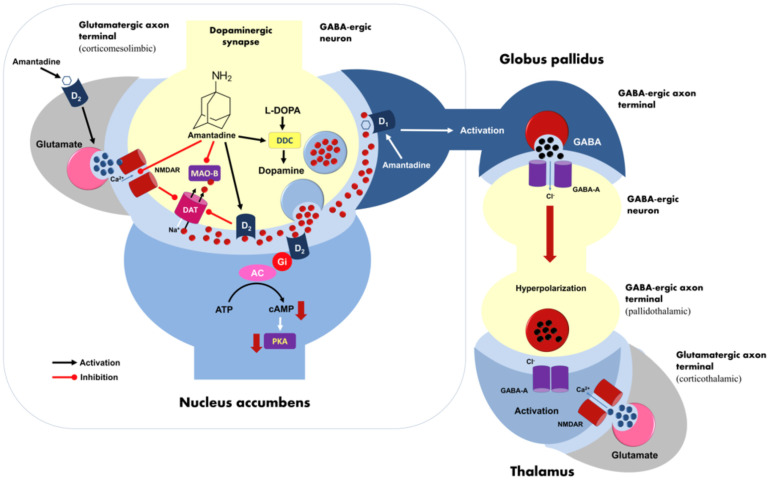
A possible neuromolecular model of amantadine action in dysthymia pharmacotherapy. Amantadine acts as a stimulator of dopaminergic signaling in the key reward system structures such as nucleus accumbens (NAc) and ventral tegmental area (VTA). It increases dopamine level within synaptic cleft due to inhibition of dopamine transporter DAT1 in NAc/VTA neurons directly and via blockage of presynaptic NMDA receptors. Additionally, it is able to inhibit monoaminoxidase B (MAO-B) but it does support presynaptic L-DOPA decarboxylase (DCC) activity. At the level of NAc neuronal circuits, amantadine may also stimulate both D1 receptors of GABAergic neurons and D2 receptors located in the corticomesolimbic glutamatergic axon terminals, increasing local glutamate release. Enhanced glutamatergic transmission may in turn elicit a further dopamine release in the NAc. Additionally, the facilitation of NAc inhibitory signaling by amantadine leads finally to the stimulation of thalamic activity. AC—adenylyl cyclase; DDC—L-DOPA decarboxylase; DAT—dopamine transporter; MAO-B—monoaminoxidase B; PKA—protein kinase A.

Amantadine stimulates mesolimbic dopaminergic pathways via blockade of dopamine transporter 1 (DAT1). This can activate DAT-controlling presynaptic D2 receptors of ventral tegmental area (VTA). Amantadine may also elicit an agonistic effect on the D1 receptors in the nucleus accumbens (NAc) [14] and act as an inhibitor of monoamine oxidase B (MAO-B). Importantly, amantadine as a noncompetitive NMDA receptor antagonist is considered a silencer of glutamatergic signaling. An inhibition of glutamatergic input disactivates DAT1 action and finally increases dopamine level in the NAc. On the other hand, amantadine may also activate D2 receptors located in the corticomesolimbic glutamatergic axonal inputs to basal ganglia, promoting glutamate exocytosis in the NAc and striatum. Elevated glutamatergic transmission may evoke a further dopamine efflux in the NAc [15]. Amantadine can also facilitate the GABA release from the NAc neurons. Their perikarya send inhibitory afferents to VTA and globus pallidus (GB). Both VTA and GB sent in turn GABAergic projection to the thalamus. Amantadine action at the level of NAc evokes finally a subsequent disinhibition of thalamic activity [14]. However, it may be suggested that NAc neural populations are regulated by both glutamatergic and GABA-ergic signaling simultaneously. The pharmacological model of dopamine receptor-related amantadine action at the level of this limbic structure may probably be more complex. A graphical summary of the mechanisms of action in amantadine is presented in Figure 1.

The efficacy of amantadine in the treatment of dysthymia is probably related to its prodopaminergic mechanism of action. Amantadine, by affecting the reward system, can improve depressive symptoms such as anhedonia, lack of drive and mental fatigue. Clinical observations and treatment of patients with dysthymia show that they often complain of anhedonia, lack of motivation and lack of drive. The relationship between dysthymia and anhedonia was noticed many years ago. They found that people with dysthymia and people with anhedonia show similar hyporesponsiveness of information processing and reduced values of the brain event-related potentials [16]. Perhaps reduction in stressor-related rewarding value of brain stimulation may combine anhedonia and depression with disturbances of the reward system. We recently published a case series study showing the effectiveness of treating bipolar depression with amantadine [12]. As in dysthymia, patients with bipolar depression often complain of anhedonia, lack of drive, and fatigue, which may in turn be associated with decreased dopaminergic activity in the reward system. This is probably why the prodopaminergic drug amantadine is effective in the treatment of bipolar depression.

Amantadine has a very complex mechanism of action, which also includes its effect on the glutamatergic system. Therefore, it cannot be ruled out that NMDA receptor blocking may also be responsible for the antidepressant effects of amantadine, as is the case with esketamine and ketamine [17]. The potential antidepressant effect of amantadine related to its anti-NMDA activity is an additional rationale for the use of amantadine in the treatment of depressive disorders.

The use of amantadine for the treatment of bipolar depression may be effective in the first week of treatment [12]. In the presented case series study, significant efficacy in the improvement of depressive symptoms in dysthymia patients was observed after the first month of treatment, which may be a favorable clinical feature of amantadine. Usually, in the treatment of dysthymia with drugs with proven effectiveness, it takes up to 3 months for improvement [4]. It is worth noting that after the first month of treatment, the improvement in the studied patients increased significantly with the extension of the treatment time.

The treatment of dysthymic patients with amantadine, despite its clinical efficacy confirmed in the pilot case series study, is an off-label use, in the sense of repurposing of the drug. The off-label use of drugs is common in psychiatry; in the group of antidepressants alone, off-label indications have been reported in up to 29% of prescribed drugs [18]. Off-label prescribing regulations differ from country to country [19]. In Poland, there is no regulation requiring the doctor to use an in-label drug, and off-label treatment does not constitute a medical experiment if the therapy is based on credible scientific evidence published in recognized scientific journals, confirming the effectiveness and legitimacy of the selected treatment procedure [20].

## 4. Materials and Methods

The study covered five cases of patients treated for dysthymia, diagnosed based on ICD-10 research criteria. The criteria for selecting the described cases into a series of cases were no improvement in at least one pharmacological treatment of an episode of dysthymia and treatment with amantadine. The medical records of patients with dysthymia treated with amantadine were reviewed, and then cases were selected for patients who had been treated for a similar period, had similar visits and were followed-up after amantadine was withdrawn. According to legal regulations in Poland, before starting amantadine, the mechanism of action of amantadine was explained to each patient and information on potential side effects was provided. Each patient consented to off-label treatment with amantadine.

Due to the retrospective nature of the collected research material, an external control group was appointed in the project. An externally controlled group allows to compare a group of subjects receiving the amantadine treatment with a group of patients external to the study (not collected at the same time). In our study, the external control was a group of patients matched for age and gender to the study group who improved with sertraline at a dose of 100 mg/d; thus, changes from baseline were compared with an estimate of what would have happened to the patients in the condition of treatment with one of the standards of dysthymia treatment. Adopting this study design (with external comparators), we have identified key factors affecting bias according to Gray et al. (2020) and we have minimized or excluded their impact [21].

The external control group consisted of five patients, two men aged 35 and 43 and three women aged 38, 41, and 36, selected after reviewing a database of patients treated for dysthymia in the past five years. The external control group was matched with respect to the sex and age of the patients and similar frequency of visits and length of treatment as in the group of patients treated with amantadine. To unify this group, sertraline was selected in a as one of the therapeutic standards in the treatment of dysthymia. The criterion for inclusion in the external control group was also achieving clinical improvement in the treatment of dysthymia when treated with the standard of care drug. Characteristics of the study and control group were presented in Table 4.

Depressive symptoms in all patients were assessed using the clinician-administered 17-item Hamilton Depression Rating Scale (HDRS-17) [22]. Symptoms were assessed prior to treatment initiation and at three subsequent visits, every 4–5 weeks. Patients received amantadine 100 mg per day in the morning. The adopted dose was based on previous studies [6,10,11,12]. The patients were routinely informed that in the event of significant side effects or worsening of depressive symptoms, they should discontinue amantadine. Amantadine was introduced the day after discontinuation of the previous drug.

The statistical analysis of obtained results was performed with the one-way repeated measures ANOVA (for the general effect of compared groups) with partial Eta-square size effect calculation. The statistical analysis in the study group was performed using Student’s *t*-test for paired data (for difference between the first and the last visit) with Cohen’s effect size and the nonparametric Friedman Test for repeated measures (for general effect) with Eta-square size effect calculation. Descriptive statistics include mean values and confidence intervals. For all analyses, the IBM SPSS v28 statistical package was used.

## 5. Conclusions

The present study indicates that amantadine is an effective and well-tolerated drug in the treatment of dysthymia. The effect of amantadine treatment is comparable to that of sertraline treatment in patients with dysthymia who improved with this drug. Amantadine may be associated with a quick improvement in symptoms in the treatment of dysthymia. Treatment with this drug seems to be associated with good tolerability and persistency of the therapeutic effect after the discontinuation of the treatment. Amantadine may become one of the drugs of choice in the treatment of dysthymic patients who have not achieved clinical improvement after previous pharmacological treatment. Confirmation of this indication requires a clinical trial with a placebo in a larger group of patients. All those preliminary conclusions must be confirmed in clinical trial in larger number of patients.

## Figures and Tables

**Table 1:** Results in individual total score in HDRS-17 scale at subsequent visits.

Visit No.	Patient 1	Patient 2	Patient 3	Patient 4	Patient 5	Mean [95% CI]	Statistical Difference
1	5	6	7	7	6	6.2 [5.6; 6.8]	t(4) = 13.42; *p* < 0.001; d = 1.0
2	3	3	3	1	3	2.6 [1.8; 3.0]	
3	0	1	0	0	1	0.4 [0.0; 0.8]	
4	0	0	0	0	1	0.2 [0.0; 0.6]	

Note: statistical difference—calculated with Student’s *t*-test for paired data (for difference between the first and the last visit) and Cohen’s effect size; 95%CI—95% confidence interval.

**Table 2:** Results in individual total score in HDRS-17 scale at subsequent visits.

Visit	Patient 1 Male		Patient 2 Female		Patient 3 Female		Patient 4 Female		Patient 5 Male		Mean [95% CI]		Statistical Difference
Group	S	C	S	C	S	C	S	C	S	C	S	C	U = 7.00; Z = −1.20; *p* = 0.230; Δ_Glass_ = 1.19
1	5.0	6.0	6.0	7.0	7.0	6.0	7.0	8.0	6.0	9.0	6.2 [5.6; 6.8]	7.2 [5.6; 8.8]	U = 6.00; Z = −1.68; *p* = 0.093; Δ_Glass_ = 0.89
2	3.0	3.0	3.0	3.0	3.0	4.0	1.0	3.0	3.0	4.0	2.6 [1.8; 3.0]	3.4 [2.7; 4.1]	U = 5.00; Z = −1.96; *p* = 0.050; Δ_Glass_ = 1.09
3	0.0	1.0	1.0	1.0	0.0	1.0	0.0	1.0	1.0	1.0	0.4 [0.0; 0.8]	1.0 [1.0; 1.0]	U = 10.00; Z = −1.00; *p* = 0.317; Δ_Glass_ = 0.45
4	0.0	0.0	0.0	0.0	0.0	0.0	0.0	0.0	1.0	0.0	0.2 [0.0; 0.6]	0.0 [0.0; 0.0]	U = 7.00; Z = −1.20; *p* = 0.230; Δ_Glass_ = 1.19

Note: statistical difference—calculated between subject, within each visit, with Mann–Whitney U test and Glass’s delta effect size; S—study group; C—control group; 95%CI—95% confidence interval.

**Table 3:** Severity of symptoms in individual items (I1–I17) of the HDRS-17 scale at subsequent visits (V1–V4) in patients (P1–P5) diagnosed with dysthymia.

Study Group									
Patient Number (M-Male, W-Female)	Visit	I-1 Depressive Mood	I-3 Suicide	I-7 Work and Activities	I-8 Retardation	I-10 Anxiety, Psychosis	I-13 General Somatic Symptoms	I-14 Genital Symptoms	Sum
P1 (M)	V1	1	0	3	0	0	0	1	5
	V2	0	0	2	0	0	0	1	3
	V3	0	0	0	0	0	0	0	0
	V4	0	0	0	0	0	0	0	0
P2 (W)	V1	0	1	3	0	0	0	2	6
	V2	0	0	2	0	0	0	1	3
	V3	0	0	1	0	0	0	0	1
	V4	0	0	0	0	0	0	0	0
P3 (W)	V1	1	0	4	0	0	0	2	7
	V2	0	0	2	0	0	0	1	3
	V3	0	0	0	0	0	0	0	0
	V4	0	0	0	0	0	0	0	0
P4 (W)	V1	1	1	3	0	0	1	1	7
	V2	0	0	1	0	0	0	0	1
	V3	0	0	0	0	0	0	0	0
	V4	0	0	0	0	0	0	0	0
P5 (M)	V1	0	0	4	0	0	0	2	6
	V2	0	0	2	0	0	0	1	3
	V3	0	0	0	0	0	0	1	1
	V4	0	0	0	0	0	0	0	0
Control Group									
Gender	Visit nr	I-1: Depressive Mood	I-3: Suicide	I-7: Work and Activities	I-8 Retardation	10 Anxiety, Psychic	I-13: General Somatic Symptoms	I-14: Genital Symptoms	Sum
P1 (M)	V1	1	0	3	2	0	0	0	6
	V2	0	0	2	1	0	0	0	3
	V3	0	0	1	0	0	0	0	1
	V4	0	0	0	0	0	0	0	0
P2 (W)	V1	1	0	2	1	1	1	1	7
	V2	0	0	1	0	1	0	1	3
	V3	0	0	1	0	0	0	0	1
	V4	0	0	0	0	0	0	0	0
P3 (W)	V1	1	0	3	1	0	0	1	6
	V2	0	0	3	0	0	0	1	4
	V3	0	0	1	0	0	0	0	1
	V4	0	0	0	0	0	0	0	0
P4 (W)	V1	1	0	3	2	0	0	2	8
	V2	0	0	2	0	0	0	1	3
	V3	0	0	1	0	0	0	0	1
	V4	0	0	0	0	0	0	0	0
P5 (M)	V1	1	1	3	2	0	0	2	9
	V2	0	0	2	1	0	0	1	4
	V3	0	0	0	0	0	0	1	1
	V4	0	0	0	0	0	0	0	0

**Table 4:** Characteristics of the study group and the control group in relation to sex, age, pharmacological treatment in the study, and year in which patients took the study drug or standard-of-care drug.

Patients	Gender	Drug	Daily Dose [mg]	Age [years]	Year of Treatment
study group case 1	male	amantadine	100	34	2020
study group case 2	female	amantadine	100	38	2019
study group case 3	female	amantadine	100	42	2020
study group case 4	female	amantadine	100	38	2019
study group case 5	male	amantadine	100	44	2021
control group case 1	male	sertraline	100	35	2018
control group case 2	female	sertraline	100	38	2019
control group case 3	female	sertraline	100	41	2022
control group case 4	female	sertraline	100	36	2021
control group case 5	male	sertraline	100	43	2022

## Data Availability

Data sharing not applicable.
